# Molecular detection and epidemiological distribution of poultry respiratory viral pathogens in commercial chicken flocks in Bangladesh

**DOI:** 10.1016/j.psj.2024.104679

**Published:** 2024-12-16

**Authors:** Md Mohi Uddin, Alamgir Hasan, Ismail Hossain, Sumyea Binta Helal, Jahan Ara Begum, Gwenaëlle Dauphin, Emdadul Haque Chowdhury, Rokshana Parvin

**Affiliations:** a Department of Pathology, Faculty of Veterinary Science, Bangladesh Agricultural University, Mymensingh 2202, Bangladesh; b Scientific Support and Investigation Platform, Ceva Animal Health, Libourne, France

**Keywords:** Molecular identification, Respiratory virus, Distribution pattern, Commercial chickens, Co-infection

## Abstract

Respiratory viral infections have a considerable detrimental impact on animal health as well as significant financial consequences in the poultry industry. The primary aim of this study is to investigate the major pathogens involved in respiratory diseases of poultry, the co-infection rate, and their epidemiological distribution in commercial chicken farms in Bangladesh. From June 2022 to December 2023, 300 pooled samples (swabs from live birds, and respiratory tissues from dead birds) were collected from the selected poultry farms where respiratory outbreaks were noticed. Samples were screened for five important respiratory pathogens circulating in Bangladesh including H9N2 low pathogenic avian influenza virus (LPAIV), H5N1 highly pathogenic avian influenza virus (HPAIV), infectious bronchitis virus (IBV), Newcastle disease virus (NDV), and infectious laryngotracheitis virus (ILTV). One-step qPCR was performed using either TaqMan probe-based or SYBR Green chemistry. A total of 140 flocks (46.67 %) were found infected with these respiratory pathogens including 18.57 % H5N1(n = 26), 20 % H9N2 (n = 28), 50 % IBV (n = 70), 37.15 % NDV (n = 52), 0.71 % ILTV (n = 1) and 26.40 % (n = 37) mixed infections were present. The present study also revealed that co-infection of several respiratory pathogens (26.40 %) caused more serious synergistic pathogenic effects and complicating factors in poultry infections. In Bangladesh, for this study, the most common season for infections in layer, broiler, broiler breeder, and Sonali is spring where these infections exhibited a consistent pattern. Nevertheless, the investigation revealed the continuous co-circulations of various respiratory viruses, resulting in a complex environment in the poultry industry. This information also helps raise farmer-level disease awareness to prevent and control the spread of viruses.

## Introduction

The commercial poultry sector in Bangladesh includes layers, broilers, and slow-growing colored meat-type chickens known as Sonali, collectively yielding approximately 0.2 million metric tons of poultry meat and an annual output of 5,210 million table eggs. A significant number (60–70 %) of commercial chickens in Bangladesh are kept in small-scale facilities with inadequate managerial practices and biosecurity controls, which increases the poultry's vulnerability to various diseases (Parvin et al., 2022).

Avian respiratory diseases pose a substantial threat to the poultry industry, causing widespread occurrence, mortality, morbidity, and economic losses while also having adverse effects on bird performance. Some infections, with zoonotic importance, further jeopardize public health. The complex nature of these diseases, involving pathogens like Avian Influenza Virus (AIV), Newcastle Disease Virus (NDV), Infectious Bronchitis Virus (IBV), and Infectious Laryngotracheitis Virus (ILTV), intensifies the challenges faced by the poultry sector (Roussan et al., 2008).

Surveillance and genetic studies indicate that H5N1 and H9N2 viruses have become endemic in Bangladesh since 2007. Virus genetic analysis is essential to monitor dynamic evolution and possible reassortment events of each subtype of AIV (Parvin et al., 2022).

IB, a key component in mixed infections, primarily affects the upper respiratory tract in chicks. Besides that, it causes decreased egg production with deformed, soft, irregular, and poor-quality eggs in breeders and layers. Some strains of IBV exhibit nephropathogenic characteristics, leading to interstitial nephritis, particularly in chicks. Despite regular vaccinations with H120, MA5, and the IB 4/91 strain, IBV continues to exert diverse effects on the poultry industry in the country (Bhuiyan et al., 2019b).

ILT is commonly found in densely populated poultry production areas, causing substantial production losses characterized by increased mortality, reduced egg production, delayed body weight gain, and increased susceptibility to other respiratory pathogens.

Newcastle Disease (ND) remains a potential threat for the poultry industry despite robust vaccination programs in numerous countries. Since its initial outbreak in 1981, it became endemic in Bangladesh and has been frequently detected and isolated from commercial poultry (Rahman et al., 2021).

Furthermore, insufficient biosecurity compliances in the Bangladeshi's poultry farms create a gateway for pathogens entry. However, proper vaccination and vaccine quality of chickens with appropriate vaccines containing prevailing strains of viruses give protection against viral diseases. In light of the recurrent appearance and transmission of respiratory pathogens within the poultry industry in Bangladesh, extensive and continuous surveillance is required to better understand the dynamics of pathogen circulation and evolution and inform on required prevention and control measures at local farms as well as broader levels. Therefore, we conducted a two-year monitoring survey spanning various regions in Bangladesh focusing on layer, broiler, broiler breeder and Sonali chickens. The objective was to detect and characterize the respiratory pathogens circulating in the country and evaluate their ongoing circulation trends. Throughout our investigation, we highlighted the emergence of five important respiratory viruses within poultry flocks in Bangladesh from 2022 to 2023.

## Materials and methods

### Ethical approval

The current study has been approved by the ethical committee of the Bangladesh Agricultural University Research System under the approval number BAURES/ESRC /ET_28122.

### Sampling frame

The study was conducted in all divisions of Bangladesh, including Dhaka, Rangpur, Rajshahi, Khulna, Barisal, Mymensingh, Sylhet, and Chattogram, from June 2022 to December 2023. For that, 23 districts (42 Upazilas) were selected, and 300 pooled samples were collected from 300 affected flocks. Among these samples, 100 were from layer, 130 were from broilers, 20 were from broiler breeders, and 50 were from Sonali chickens. The farms were selected randomly based on the density of the poultry farms and upon confirmation of at least two respiratory signs (respiratory distress, nasal discharge). In this study, the season is divided into four categories: summer (June to August), fall (September to November), winter (December to February) and spring (March to May). Additionally, the age groups of birds were divided into different categories: Sonali (0–4, >4 weeks), broiler (0–2, >2 weeks), layer and broiler breeder (0–6, 6–18, >18 weeks). The on-farm inquiry, detailed records of the disease history, and observed clinical signs were documented (available on request). Farms with birds exhibiting clinical signs or necropsy findings suggestive of respiratory infections were chosen for virological investigation. For dead birds, necropsy was conducted by cervical dislocation by a trained veterinarian with appropriate safety measures, and any visible pathological changes were noted. Respiratory tissues (lungs and trachea) were aseptically collected in sterile screw-capped tubes, and oropharyngeal swabs, cloacal swabs, and fecal samples were obtained from live birds showing respiratory symptoms using a sterile phosphate-buffered saline (PBS) solution supplemented with antibiotics and stored at −80 °C. A total of five swab samples were gathered from each farm and combined into a pooled sample.

### Nucleic acid extraction

To detect the viral presence, trachea and lung tissue samples, or swabs, were placed in 2 mL of minimal essential medium containing penicillin and streptomycin. Each organ sample was homogenized with a single 5 mm stainless steel bead in a 2 mL collection tube for 2 min using a Tissue Lyser instrument (Qiagen, Hilden, Germany). After centrifuging the tissue samples, 200 μl of the resulting supernatant was carefully collected into individual Eppendorf tubes. Nucleic acid extraction was carried out using the GeneJET Viral DNA/RNA Purification Kit (Thermo Fisher Scientific, USA) for the RNA viruses and DNeasy Blood & Tissue Kit (Qiagen, Germany) for the DNA virus following the provided kit instructions.

### qPCR and RT-qPCR for DNA and RNA amplification

The presence of H5 HPAI, H9 LPAI, NDV, IBV, and ILTV in the samples was detected by RT-qPCR and qPCR using the previously published gene-specific primers and probes for the respective pathogens (Parvin et al., 2022). In the case of AIV, the AgPath Universal Probe One-Step RT-qPCR Kit (Thermo Fisher Scientific, USA) was employed for the M gene. Further, all AIV-positive samples were tested for H5, N1, H9 and N2 genes by the same procedure. The final reaction volume was 12.5 μl μl, containing 2.5 μl RNA template, 6 μl of 2 × RT-qPCR reaction mix, 0.5 μl of RT- PCR Enzyme Mix, 1.5 μl of nuclease-free water, and 2 μl of primer-probe mix (10 pmol each). The RT-qPCR thermocycling conditions were 45 °C °C for 10 min (reverse transcription) and 95 °C for 10 min (initial denaturation), followed by 40 cycles at 95 °C for 15 s (denaturation) and 60 °C for 1 min (annealing and elongation) with the reading of fluorescence in this step. After completion of the assay, the amplification curves were viewed and the cycle threshold (Ct) value was recorded. The cycle threshold (Ct) value was used to semi-quantify the viral load and a Ct value of <35 was used as a threshold for positive results.

The detection of NDV, IBV, and ILTV targeted the NP gene, IBV UTR region, and ILT glycoprotein gene-specific oligonucleotides, respectively. Luna Universal One-Step RT-qPCR Kit (New England Biolabs Inc., USA) containing SYBR Green reagents was used following a previously standardized protocol (Nooruzzaman et al., 2021). The final reaction volume was 12.5 μl, including 2.5 μl of the RNA template, 5 μl of 2 × RT-PCR SYBR reaction mix, 0.5 μl of Luna WarmStart RT Enzyme Mix, 2.5 μl of nuclease-free water, and 2 μl of primer mix (10 pmol each). The RT-qPCR thermocycling conditions were 55 °C for 10 min (reverse transcription) and 95 °C for 2 min (initial denaturation), followed by 45 cycles at 95 °C for 10 s (denaturation) and 60 °C for 30 s (annealing and elongation) with a reading of fluorescence in this step. As ILTV is a DNA virus we performed qPCR using the same kit excluding the reverse transcription step. Immediately after PCR, a melting curve analysis was performed with a continuous temperature increment of 0.5 °C/s between 65 and 95 °C. After completion of the assay, the amplification curves were viewed and the cycle threshold (Ct) value and melting temperature (Tm) were recorded.

### Statistical analysis

An UpSetR plot, was employed to visually represent co-occurrences among respiratory pathogens. This online platform is known for effectively illustrating sets of co-occurring variables and displaying their intersections through a bar chart. The rest of the data analysis and graph preparation was conducted using GraphPad Prism 8.0.

## Results and discussion

### Overall occurrence of avian respiratory pathogens in poultry flocks

AIV, NDV, IBV, and ILTV tests were performed on 300 poultry flocks with a history of respiratory symptoms, mortality, and gross lesions (Table 1), and 140 flocks tested positive. The overall occurrence of respiratory diseases in poultry was found to be 46.67 % (n = 300). Among different poultry types, broiler breeders showed a slightly lower occurrence of 42.85 %, followed closely by layer birds at 53 %, broiler chickens exhibited the occurrence of 62.01 %, while Sonali chickens demonstrated the highest occurrence at 70 %. Among all pathogens, IBV was the most prevalent at 50 %, followed by NDV at 37.15 %, H9N2 at 20 %, H5N1 at 18.57 %, and ILTV at 0.71 %. Mixed viral infections were observed in 26.40 % of cases with various combinations. For many years, Bangladesh has reported a prevalence of respiratory infections in commercial poultry ranging from approximately 30–50 % (Nooruzzaman et al., 2021), consistent with our findings.

**Table 1:** Summary of investigated poultry flocks and their positive agents in the current study.

Bird type		Layer	Broiler	Broiler Breeder	Sonali
Number of flocks		100	130	20	50
Flock size range		1000–28000	1000–4000	3000–11155	1000–155000
**Vaccination status**	**H5N1**	(49 flocks vaccinated) Vectormun HVT AIV, Avieza vac H5, Reassortant avian influenza virus, Vectormun AI	Not vaccinated	(10 flocks vaccinated) Vectormun HVT-AI, H5N1-RE6	(6 flocks vaccinated) Reassortant avian influenza virus, Vectormune HVT-AI
	**H9N2**	(61 flocks vaccinated) Cevac new Flu h9 K, Advance (Not known), Avienza vac H9, Nobilis H9N2, Vaxxon Aviflu	Not vaccinated	(8 flocks vaccinated) New Flu H9k	(3 flocks vaccinated) Cevac new Flu H9k
	**NDV**	(99 flocks vaccinated) Cevac BIL, Innovax ND+IBD, Hipraviar ND+IB, BCRDV+RDV, Nobilis MA5+clone 30, Bangla BCRVD, Bangla ND+IB, Cevac BIL+Cevac new L, RDB, Izovac ND+IBD, Poulshot NDO, Ranivax Plus, Poulshot lasota, Izovac B1 Hitchner, Izovac clone, Nobilis MA5+clone 30+Hipraviar Clin/79, Nobilis ND lasota, Nobilis Newcevac	(113 flocks vaccinated) BCRDV+RDV, Innovax ND+IB, Cevac BIL, Poulshot lasota, Bangla BCRDV, Hipraviar ND+IB, Nobilis MA5+clone 30, Ranivax Plus initial, Cevac BIL+Cevac new L, Immugal VP lasota, ITA ND+IBD, Bangla ND vac, Cevac new K, Vectormun ND, Ranivax booster	(18 flocks vaccinated) Nobilis MA5+clone 30, Cevac BIL, Cevac new L	(44 flocks vaccinated) Bangla BCRDV, Cevac BIL, Cevac new L, Izovac B1, Hipraviar BPL2, Hipra 49, Innovax ND+IBD, Nobilis clone 30, MSD+Ceva live and killed, Avinew
	**IBV**	(89 flocks vaccinated) Cevac BIL, Hipraviar ND+IB, Innovax ND+IBD, Cevac Ibird, Bangla ND+IB, Nobilis MA5, Nobilis MA5+Nobilis IB+ND, Ranivax plus initail	(54 flocks vaccinated) Innovax ND+IB, Cevac ibird, Hipraviar ND+IB, Nobilis MA5, Bangla ND+IB, Ranivax plus initail, Nobilis IB4/91	(12 flocks vaccinated) Cevac Ibird, Nobilis IB4/91	(34 flocks vaccinated) Bangla ND+IB, Cevac Ibird, Hpraviar B1/H120, Nobilis MA5, Cevac BIL
	**ILTV**	Not vaccinated	Not vaccinated	Not vaccinated	Not vaccinated
**Clinical signs**		Gasping, sneezing, coughing, respiratory distress, nasal and ocular discharge, cough in mouth, snoring sound, drowsiness, whitish to greenish feces, diarrhea, off feed, weakness, deformed egg, certain drop in egg production, sudden death	Gasping, sneezing, coughing, ruffled feathers, depression, off-feed, sudden death, weakness.	Penguin-like posture and high mortality, coughing, respiratory distress, nasal and ocular discharge, and certain drops in egg production	Respiratory distress, depression and off-feed, torticolis, sneezing, coughing, paralysis, penguin posture, greenish droppings
**Mortality** **average (%)**		7.34	3.57	1.49	3.68
**Major necropsy** **findings**		Congestion in trachea and lung; cloudy air sac, highly fragile liver, hemorrhages in cecal tonsil, intestine, pancreas, muscle, liver, brain, and gizzard; hemorrhage on proventriculus and base of the heart	Hemorrhages in muscle, congestion in the trachea and lung, cloudy air sac, ascites; hydropericardium, fragile liver	Hemorrhages in muscle, congestion in the trachea and lung, cloudy air sac, ascites; hydropericardium, fragile liver, hemorrhage on proventriculus and base of the heart	Congestion in the trachea, lung, cloudy air sac, liver, intestine and mesentery; mottled spleen
**Positive agents**		H5 HPAI (n = 8) H9 LPAI (n = 10) IBV (n = 16) NDV (n = 18) ILTV (n = 1) Mixed (n = 10)	H5 HPAI (n = 7) H9 LPAI (n = 14) IBV (n = 36) NDV (n = 23) Mixed (n = 15)	H5 HPAI (n = 3) H9 LPAI (n = 2) IBV (n = 3) NDV (n = 1) Mixed (n = 3)	H5 HPAI (n = 8) H9 LPAI (n = 2) IBV (n = 15) NDV (n = 10) Mixed (n = 9)

### Regional occurrence of avian respiratory pathogens

Between June 2022 and December 2023, we gathered 300 flock samples from layer, broiler, broiler breeder, and Sonali. Among these, 60 samples were obtained from Dhaka, 50 from Rajshahi, 40 each from Mymensingh and Rangpur, and 30 each from Barisal, Khulna, and Chattogram, with an additional 20 collected from Sylhet.

**Figure 1:**
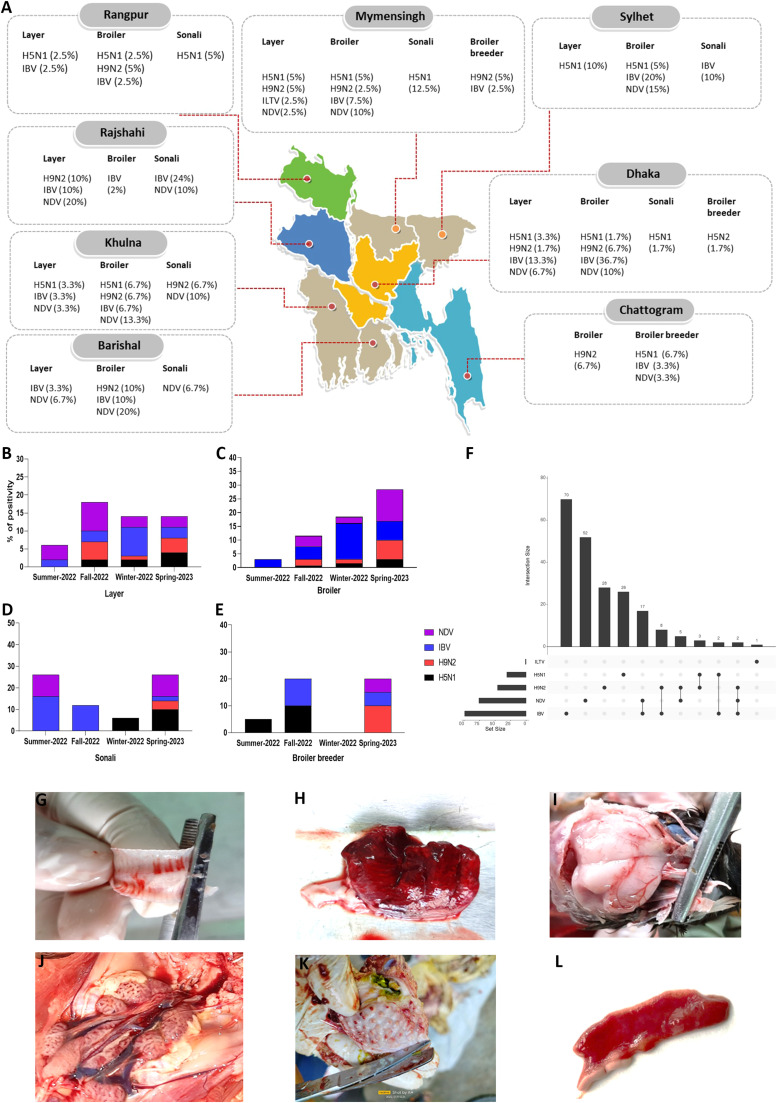
Detailed analysis of respiratory pathogens occurrences in diverse poultry types across different regions of Bangladesh from June 2022 to December 2023 (Fig. 1A). Seasonal distribution of avian respiratory pathogens (June 2022–December 2023): The *X*-axis denotes seasons (Summer, Fall, Winter, Spring), while the *Y*-axis illustrates the percentage of positive samples (Fig. 1B–E). An UpSetR plot showing co-occurrences of 5 respiratory pathogens in layer, broiler, broiler breeder and Sonali farms. Here, ‘Set Size’ denotes the combinations of co-occurring pathogens and the ‘Intersection Size’ indicates the frequency of each set or combination. H5N1, highly pathogenic avian influenza virus; H9N2, low pathogenic avian influenza virus; NDV, Newcastle disease virus; IBV, infectious bronchitis virus, ILTV, infectious laryngotracheitis virus (Fig. 1F). Gross pathological lesions in affected chickens show severe congestion and hemorrhage in the lung, mild to severe hemorrhages in the trachea, brain, kidney, proventriculus, and intestine across different poultry species (Fig. 1G–L).

In the assessment of different regions, in layer, it was observed that the occurrence of H5N1 was most pronounced in the Sylhet region, reaching 10 %. Meanwhile, in the Rajshahi district, the occurrence of H9N2 and NDV peaked at 10 % and 20 %, respectively. Notably, Dhaka emerged with the highest occurrence of IBV at 13.3 %. Regarding broiler sample analysis, H5N1 exhibited its highest occurrence in the Khulna region, recording an occurrence of 6.7 %. Meanwhile, in the Barishal district, the occurrence of H9N2 and NDV reached their peak at 10 % and 20 %, respectively. A noteworthy finding was the significant occurrence of IBV in Dhaka, where it peaked at 36.7 %. Mymensingh region had the highest frequency (12.5 %) of H5N1 in Sonali. Meanwhile, in the Khulna district, the occurrence of H9N2 and NDV peaked at 6.7 % and 10 %, respectively. A significant finding was the highest occurrence of IBV in Rajshahi, reaching 24 %. In broiler breeders, the occurrence of H5N1, NDV and IBV was most pronounced in the Chattogram region, reaching 6.7 %, 3.3 % and 3.3 % respectively. Meanwhile, in the Mymensingh district, the occurrence of H9N2 peaked at 5 % (Fig. 1A).

### Seasonal dynamics of respiratory pathogens across poultry types

Within the layer category, the occurrence of NDV and H9N2 was notably higher at 8 % and 5 %, respectively, during the fall season compared to other seasons. In contrast, IBV was more frequent (8 %) in the winter season, and H5N1 exhibited a higher occurrence (4 %) during the spring season (Fig. 1B). During the spring season, the occurrence of three pathogens (H5N1, H9N2, and NDV) in broilers was notably higher, with percentages of 3.07 %, 6.92 %, and 11.53 %, respectively. In contrast, the winter season recorded the highest incidence of IBV at 13.07 % (Fig. 1C). In the Sonali bird species, the occurrence of respiratory pathogens, specifically H5N1, H9N2, and NDV, was approximately 10 %, 4 %, and 10 %, respectively, showing a similar pattern to broiler birds. Additionally, IBV had a higher occurrence of 16 % during the summer season (Fig. 1D). In broiler breeders, despite the limited sample size, the occurrence of H5N1 and IBV was significantly higher at 10 % each during the fall season. The other two pathogens, H9N2 and NDV, showed higher frequencies of 10 % and 5 % during the spring season (Fig. 1E). Overall, the peak seasons for H5N1, H9N2 and NDV were in the spring season and IBV occurred throughout the year but peaked in the winter season. Some observations corresponded with our findings such as a small peak in avian influenza cases observed in April (Berry et al., 2022), and a high incidence of IBV noted during the winter season (Ali, 2020). However, the findings of Al Mamun et al. (2019) reported a high incidence of NDV in the winter season (Al Mamun et al., 2019), which does not align with our results.

### Age-related patterns of respiratory pathogens in poultry flocks

In the 0–4 weeks age range, Sonali chickens displayed the highest percentage for IBV at 8 %, which was consistent with a similar occurrence beyond 4 weeks of age where the percentage increased to 22 %. Additionally, the occurrence of NDV was 6 % in the 0–4 weeks age group and increased to 14 % in the >4 weeks of age category. In Broiler chickens, the highest occurrence of IBV and NDV was observed before 2 weeks of age, reaching 6.93 %. Beyond 2 weeks of age, a consistent pattern was observed with positivity rates of 20.77 % for IBV and 10.77 % for NDV. In Layer chickens, the occurrence of NDV peaked at the brooding stage (0–6 weeks), reaching 3 %. During the growing stage (6–18 weeks), IBV became more prominent, with an occurrence of 4 %. Beyond 18 weeks, the percentages increased to 7 % for H5N1, 8 % for H9N2, 10 % for IBV, 14 % for NDV, and 1 % for ILTV. For Broiler Breeders aged over 18 weeks, the percentages of positive samples were 15 % for H5N1, 10 % for H9N2, 15 % for IBV, and 5 % for NDV. According to the age, we observed that the occurrence of these respiratory viruses increased with increasing age. Other studies also noted higher age of chickens are prone to IBV and ILTV infection (Rahman et al., 2018).

### Co-occurrence patterns of different respiratory pathogens

Co-infections are represented in Fig. 1F as an UpSetR plot, where sets are the different combinations of the co-occurrences of pathogens and intersection bars are the frequencies of different combinations. Co-occurrence of 2 pathogens was found in 35 samples with 5 different combinations, IBV + NDV positive for 17 samples, IBV + H9N2 for 8 samples, NDV + H9N2 for 5 samples, H5N1 + H9N2 for 3 samples, IBV + H5N1 for 2 samples and co-occurrence of 3 pathogens IBV + NDV + H9N2 positive for only 2 samples. Among the five tested pathogens, the occurrence of IBV and NDV was the most frequent. Either IBV or NDV or both also occurred concurrently with other pathogens. Other reports of co-infections with multiple respiratory pathogens in poultry, involving different combinations, have been documented in various studies (Hassan et al., 2021).

### Commonly observed clinical signs and necropsy findings

In this study, the disease severity varied from flock to flock, and clinical features included gasping, sneezing, tracheal rales, listlessness, nasal discharges, and dull with ruffled feathers (Table 1). At necropsy, the common gross pathological features observed were mild to severe hemorrhages in the trachea, brain, kidney, intestine, and proventriculus (Fig. 1G–L). Congested, consolidated, and fibrinous lungs were common in most birds. In a few cases, congested spleen, thymus atrophy, and swollen and hemorrhagic bursa were observed indicating immunosuppression of the bird in the respective flocks.

## Disclosures

The authors declare that they have no known competing financial interests or personal relationships that could have appeared to influence the work reported in this paper.
